# Supplementary material for: Quantifying tobacco and alcohol imagery in Netflix and Amazon Prime instant video original programming accessed from the UK: a content analysis
Source: BMJ Open. 2019 Feb 13;9(2):e025807. doi: 10.1136/bmjopen-2018-025807 (PMC6398653; doi:10.1136/bmjopen-2018-025807)
Supplement: Supplementary file 1 [file bmjopen-2018-025807supp001.pdf]

Supplementary Table 1: Programmes coded as part of the content analysis

	Programme	Age rating[1]	Genre[2]	Episodes Coded
**Netflix**	The Crown	15	Drama	Season 1<br>Episodes 1,3,5,7 & 10
	Marvel's Daredevil	15	Action	Season 2<br>Episodes 1,4,8,10 & 13
	House of Cards	15	Drama	Season 4<br>Episodes 1,4,8,10 & 13
	Narcos	15	Biography	Season 2<br>Episodes 1,3,5,7 & 10
	Stranger Things	12	Drama	Season 1<br>Episodes 1,2,4,6 & 8
**Amazon Prime Instant Video**	Bosch	15	Crime	Season 2<br>Episodes 1,3,5,7 & 10
	Goliath	15	Drama	Season 1<br>Episodes 1,2,4,6 & 8
	Good Girls Revolt	15	Drama	Season 1<br>Episodes 2,4,6,8 & 10
	The Grand Tour	12	Comedy	Season 1<br>Episodes 1,2,4,6 & 8
	The Man in the High Castle	15	Drama	Season 2<br>Episodes 1,3,5,7 & 10

[1]According to the British Board of Film Classification (BBFC, http://www.bbfc.co.uk/)
[2]According to the Internet Movie Database (IMDB, https://www.imdb.com)
